# The impact of environment on college students' entrepreneurial intention based on the mediating effect of personality traits

**DOI:** 10.3389/fpsyg.2022.972992

**Published:** 2022-12-20

**Authors:** Jiping Jiang, Jiapan Xu, Xiaocui Yin, Jinyan Hu

**Affiliations:** ^1^Department of Education Science, Nanjing Normal University, Nanjing, China; ^2^Department of Information and Engineering, Henan Institute of Science and Technology, Xinxiang, China; ^3^School of Physical Education and Humanities, Nanjing Sport Institute, Nanjing, China; ^4^Faculty of Education, Henan Normal University, Xinxiang, China

**Keywords:** entrepreneurial intention, college students, environment factors, personality traits, mediating effect

## Abstract

**Introduction:**

The entrepreneurship rate of Chinese college students is relatively low. This study investigates the environment factors that influence college students' entrepreneurial intention as mediated by personality traits.

**Methods:**

According to the entrepreneurial ecosystem theory, a hierarchical model of the environment factors was established which included three layers: personality traits as the micro system, family and education as the meso system, and social and policy support as the macro system. The structural equation model of the environment factors was constructed to reveal the significant influence path of various factors and the mediating role of personality traits. Data were collected from 436 undergraduate students in Henan Province, China.

**Results:**

Findings indicate that family, education, social, and policy factors have no significant direct influence on entrepreneurial intention. However, personality traits significantly influence entrepreneurial intention and mediate the effects of various factors on entrepreneurial intention.

**Discussion:**

Suggestions such as improving college students' entrepreneurial practice and promoting college students' proactive personalities are also put forward.

## Introduction

Extant entrepreneurship studies agree that some characteristics of college students indeed promote their entrepreneurial intention such as self-efficacy (Arshad et al., 2016), risk-taking (Zhang et al., 2015), positive attitude (Yang et al., 2015), and others. The entrepreneurial ecosystem theory, which stems from the social ecosystem theory, attaches great importance to the interaction between humans and the environment and the effect of the environment on individual behavior. The theory primarily emphasizes the joint influence of family, education, and society on individual growth and behavior (Davies et al., 2005). However, there remains a lack of research application research of this thought on the field of entrepreneurship. The environment is rarely studied as the context factor of entrepreneurship (Bignotti and Roux, 2016). Cornerstone studies on the entrepreneurial environment include the five-dimension model proposed by Gnyawali and Fogel (1994) and the global entrepreneurship observation (GEM) study (Amorós and Bosma, 2014). Despite this, mechanisms explaining how the external environment affects the entrepreneurial intention largely remains understood as a “black box”; many even doubt whether entrepreneurship can be feasibly taught.

“Mass entrepreneurship and innovation” has become an important driving force of Chinese economic reform. However, only a very small fraction (0.4%) of Chinese college graduates started their own businesses in 2008. This has led to the Chinese Ministry of Education vigorously promoting innovation and entrepreneurship education and the entrepreneurial activities of college students since 2010 (Ministry of Education of the People's Republic of China, 2010). Consequently, the entrepreneurship rate increased to 2.9% (Mycos Institute, 2017). The entrepreneurship rate of Chinese college students also remains very low, especially given the negative impact of COVID-19 in early 2020. In 2021, 1.2% of undergraduate students (Mycos Institute, 2022a) and 3.1% of vocational college students (Mycos Institute, 2022b) chose to start their own businesses. This is alarmingly low compared with the global average entrepreneurship rate of college students of about 20%.

Thus, it is necessary to reflect on the effect of environmental factors on entrepreneurial intention. Some studies only analyze one or several factors, but there remains a lack of a reliable framework between the factors. Although some scholars have divided the influencing factors into different layers (Bronfenbrenner, 1979; Zastrow and Kirst-Ashman, 2012), the influence of the environment on entrepreneurial intention remains to be given commensurate scholarly attention. Therefore, this study attempts to explore the mechanism of environmental factors on the entrepreneurial intention of Chinese college students during the outbreak of COVID-19 using a structural equation model. It does so by dividing the environment into three layers: micro-system, meso-system, and macro-system. The micro-system refers to the individual in this system such as personality traits in the entrepreneurial environment. The meso-system refers to the small group system such as the family and education. Finally, the macro-system refers to the broader cultural and social system such as social support and policy support.

## Literature review

### Entrepreneurial intention

Entrepreneurship is the process of creating valuable new things (Hisrich and Peters, 1998). Bird (1988) was the first to propose the concept of “entrepreneurial intention” where individuals pay attention to or even commit to setting up a new company or realizing the new value of an existing company. Bae et al. (2014) defined it as an individual's desire to find a business, while Phan et al. (2002) defined it as the possibility of choosing to launch a business.

Entrepreneurial intention (EI) is often expressed through entrepreneurial willingness or entrepreneurial tendency. It is seen as the subjective attitude of individuals to devote themselves to entrepreneurial practice. Entrepreneurial intention is also the premise of entrepreneurial behavior (Krueger and Brazeal, 1994) and subsequently plays a crucial role in predicting entrepreneurial behavior.

### Influencing factors

As the premier environment is connected to the cultivation of college students' personality traits, both family support and education support can have direct impacts on them.

#### Family support

College students whose parents own companies or industries have a higher entrepreneurial intention (Gimenez et al., 2015). Some studies also argue that family plays a decisive role in the prediction of entrepreneurial intention (Zhou et al., 2021). Stronger family entrepreneurial atmospheres mean higher entrepreneurial willingness of college students (Wei and Li, 2013).

#### Education support

According to the theory of planned behavior (TPB), entrepreneurial learning affects the individual's interpretation of external information, which may lead to different entrepreneurial attitudes (Hill, 1977). Entrepreneurial knowledge and skills are positively correlated with entrepreneurial perception and willingness (Soomro and Shah, 2022). Entrepreneurial intention is, therefore, positively affected by the success of the parents' business and by parents acting as role models for their children (Davide et al., 2021). Entrepreneurship education in American universities is categorized into three groups: the education about enterprise, education for enterprise, and education in enterprise (Jamieson, 1984). There are also various entrepreneurial models, including magnet mode, radiation mode, and mixed mode. Currently, entrepreneurship education remains to be the primary mode in China, which is mainly based on the entrepreneurship theory.

#### Personality traits

Existing studies have shown that personality traits are significantly related to entrepreneurial intentions (Heydari et al., 2013). Entrepreneurial intention depends on feasibility cognition, individual action power, risk appetite, opportunity awareness, and perceived capabilities (Shapero and Sokol, 2009; Noguera et al., 2013; Tsai et al., 2016). Personality traits are pivotal as to whether an individual can become an entrepreneur (Hu et al., 2018). However, Gartner (1985) showed no significant correlation between personality traits and entrepreneurial intention, while Wagner and Sternberg (2004) showed that entrepreneurial intention is influenced by both personality traits and environmental factors.

#### The mediating role of personality traits

Studies have focused on the direct effect of various factors on entrepreneurial intention. Nonetheless, the intermediate mechanism of how they transform to entrepreneurial performance remains to be thoroughly studied, which has been regarded as a “black box” (Maritz and Brown, 2013). Proactive personality, as first proposed by Bateman and Crant (1993), is a stable tendency for individuals to proactively influence their surroundings. College students with higher levels of proactive personality identify entrepreneurial opportunities more actively, seek alliances and supporters to support individual initiative behavior, contact with influential and high-quality resources, pursue innovative strategies, and obtain better financing (Thompson, 2005). This allows them to develop the ability to quickly identify, obtain, and use external resources (Hulsink et al., 2008). Accordingly, entrepreneurial self-confidence and entrepreneurial ability are improved, in turn urging them to take positive actions, make use of the identified opportunities, realize entrepreneurship, and seek entrepreneurial success (Krueger and Brazeal, 1994). Hence, this study posits the existence of a mediating role of personality traits between entrepreneurial factors and entrepreneurial intention.

In addition, as the indirect environmental factors, entrepreneurship policies and social support also impact personality traits, further affecting entrepreneurial intention.

#### Entrepreneurship policy

Entrepreneurship policies stimulate the entrepreneurial spirit of a country or economic entities of a region and improve their entrepreneurial activities (Dahlstrand and Stevenson, 2010). Hence, positive entrepreneurial policies provide better entrepreneurial resource support (Benschop, 2010) and effective financing channels (Xiang and Sun, 2021). However, as external factors of the environment, entrepreneurship policies influence college students' internal characteristics to effectively promote their entrepreneurial intention.

#### Social support

Social support mainly demonstrates the social environment and the support provided by multiple subjects in the society from the perspective of social capital Caplan, 1976; Younis et al., 2020, meeting the needs of college students' achievement intentions and their corresponding values. The access and availability of resources are important external factors (Wagner and Sternberg, 2004; Tang, 2013), and a related culture of failure tolerance mobilizes the enthusiasm for individual innovation and entrepreneurship and promotes their participation in entrepreneurial activities.

## Conceptual framework

In its early stages, the theory of entrepreneurial event was included in the literature on entrepreneurial intention (Shapero, 1984). Then, the TPB was brought into consideration, followed by the formation of a myriad of models (Ajzen, 1991). The entrepreneurial ecosystem reveals the impact of family, university, and society on the entrepreneurial intention of college students (Davies et al., 2005). However, the influence mechanism of the environment on entrepreneurial intention is blurry. Zastrow and Kirst-Ashman (2012) divided the influencing factors into three layers: macro-, meso-, and micro-systems. The study followed Zastrow et al.'s method to divide the influencing factors of entrepreneurial intention (EI). The micro-system includes personality traits (PT). The meso-system includes family support (FS) and education support (ES), promoting the entrepreneurial intention directly, and the macro-system includes policy support (PS) and social support (SS), thus indirectly influencing the entrepreneurial intention. Moreover, personality traits are proposed to play an intermediary role.

**Figure 1:**
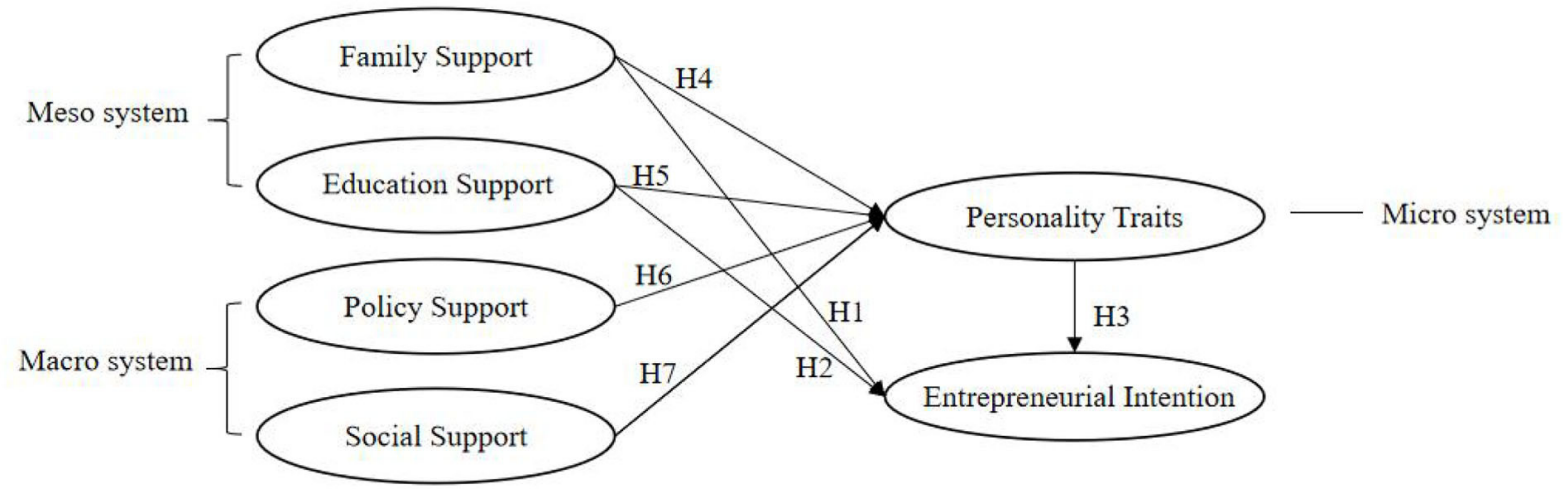
Conceptual model.

Based on the existing research, this study constructs the conceptual model. By the structural equation model, this study establishes the path of various factors on the entrepreneurial intention of college students to analyze the significant influencing factors, as shown in Figure 1. Furthermore, several research hypotheses are put forward.

Hypothesis 1: Family support has a significant impact on college students' entrepreneurial intention.
Hypothesis 2: Entrepreneurship education has a significant positive impact on college students' entrepreneurial intention.
Hypothesis 3: Personality traits have a significant impact on college students' entrepreneurial intention.
Hypothesis 4: Personality traits play a mediating role between family support and entrepreneurial intention.
Hypothesis 5: Personality traits play a mediating role between entrepreneurship education and entrepreneurial intention.
Hypothesis 6: Personality traits play a mediating role between policy support and entrepreneurial intention.
Hypothesis 7: Personality traits play a mediating role between social support and entrepreneurial intention.

## Methods

### Sample and data

Data were collected through questionnaires, the online version of which was distributed to undergraduate students in Henan Province, China, following convenience sampling. A total of 436 valid questionnaires were distributed, and the rate of reclamation was 98.9%, with 264 men (60.6%) and 172 women (39.4%). This survey involved people from various disciplines, such as natural sciences, engineering, agriculture, medicine, economics, management, law, history, education, history, and arts. The survey spanned from March 2020 to June 2020.

According to the survey data, 3.7% of the participants were pursuing a business, which partially coincides with the statistics of Mycos Institute (2022b); 56.0% of the college students have considered entrepreneurship, while 25.0% of the students only stay at the idea level but will not try. However, 15.3% had no entrepreneurial intention at all.

### Variable measurement

This study employs six variables: (entrepreneurial intention), policy support, social support, family support, education support, and personality traits. The questionnaire was designed as a five-point Likert scale ranging from 1 (strongly disagree) to 5 (strongly agree).

(1) Dependent variable—(entrepreneurial intention): Three items from Liñán and Chen (2010) were applied according to the characteristics of Chinese college students: “I am determined to create a firm in the future,” “I have very seriously thought of starting a firm,” and “I have the firm intention to start a firm some day.”

(2) Independent variables: Four independent variables were designed following Zastrow and Kirst-Ashman (2012) frameworks and other related extant studies. Entrepreneurship policies included an incubation policy (Schwarz et al., 2009), loan discount policy, industrial and commercial tax relief policy, tax relief policy, site fee reduction policy, and project funding policy. Social support includes social investment, public opinion environment, technology environment, and economic environment (Pereverzeva, 2015). Family support included parental support, parents' own business, friends' support, friends' own business, and local cultural support (Koch et al., 2015). Education support includes knowledge, skills, entrepreneurship courses, entrepreneurial projects, and teacher encouragement (Nowiński et al., 2017).

(3) Mediating variable (personality traits): Following Ismail et al. (2015), items on personality traits were further modified and consisted of three factors: innovativeness, proactiveness, and risk-taking.

### Questionnaire design and data analysis

To ensure questionnaire quality, the first draft was designed following student interviews and expert interviews. A small-scale test was conducted, and reliability and validity were analyzed. Then, the questionnaire was revised and tested repeatedly until it reached high reliability and validity. The final questionnaire was formed and implemented on a large scale.

Structural equation modeling (SEM) was used to analyze the influence factors of college students' entrepreneurial intention, which are difficult to observe directly. Partial least squares structural equation modeling (PLS-SEM) was used to predict and explain the factors through path modeling and confirmatory factor analysis. SPSS 23.0 was used to analyze the reliability and validity of the questionnaire.

## Results

### Descriptive statistics

Principal component analysis was performed in the exploratory factor analysis. The KMO value of the data is 0.929, and the significance coefficient of Bartlett's sphericity test is 0.000, indicating that it is suitable for factor analysis (Wu, 2000). The factor analysis is shown in Table 1, and six factors are obtained after rotation; the cumulative contribution rate of exceeding variance is 68.13%, and the load value of each observation variable is higher than 0.7, with at least three items per factor (Hair et al., 2010).

**Table 1:** Rotated component matrix (*N* = 436).

**Variables**	**Items**	**Description**	**Load value**	**Cronbach alpha**
Policy support	X11	Incubation policy	0.903	0.925
	X12	Loan discount policy	0.902	
	X13	Industrial and commercial tax relief policy	0.864	
	X15	Site fee reduction policy	0.853	
	X14	Tax relief policy	0.832	
	X16	Project funding policy	0.753	
Social support	X22	Public opinion environment	0.849	0.849
	X23	Technology environment	0.827	
	X24	Economic environment	0.810	
	X21	Social investment	0.803	
Family support	X31	Parental support	0.829	0.835
	X34	Friends' own business	0.795	
	X32	Parents' own business	0.778	
Education support	X44	Entrepreneurial projects	0.830	0.834
	X41	Entrepreneurial knowledge	0.786	
	X42	Entrepreneurial skills	0.750	
	X43	Entrepreneurial courses	0.667	
Personality traits	X53	Spirit of adventure	0.786	0.806
	X54	Planning and organizational capabilities	0.779	
	X51	Communication skills	0.755	
	X52	Innovation ability	0.733	
Entrepreneurial intention	X63	Determination	0.812	0.781
	X64	Serious thought	0.794	
	X61	Firm intention	0.680	

For reliability, Cronbach's alpha coefficient of the total questionnaire is 0.925, and the value of every factor is higher than 0.70 (Hair et al., 2010). The structural reliability and validity of the questionnaire are confirmed.

Discriminant validity for each variable was examined to test convergent validity. Cross-loading value criteria should be higher than 0.70 (Hair et al., 2010). The result is shown in Table 2. It can be seen that the variables of PS, SS, FS, ES, PT, and EI have a cross-loading value of upper 0.70, and every AVE is higher than 0.5. The results indicate the variables have good convergence validity.

**Table 2:** Descriptive statistics and structural validity of the scale (*N* = 436).

**Dimension**	**Mean**	**SD**	**PS**	**SS**	**FS**	**ES**	**PT**	**EI**
PS	2.221	0.880	**0.727**					
SS	2.160	0.832	0.651^**^	**0.676**				
FS	2.383	0.828	0.472^**^	0.494^**^	**0.642**			
ES	2.112	0.616	0.299^**^	0.259^**^	0.171^**^	**0.579**		
PT	1.903	0.803	0.494^**^	0.468^**^	0.403^**^	0.054	**0.583**	
EI	1.934	0.872	0.478^**^	0.470^**^	0.451^**^	0.083	0.743^**^	**0.584**

** *p* < 0.01. Bold values are the square root of the AVE of each construct. Off diagonals are Pearson correlation of constructs.

### Fitting analysis of the structural model

According to the research hypothesis, AMOS 24.0 is used to establish a structural equation model for path analysis to further analyze the influence of various factors on the entrepreneurial intention. In the fitting index of a structural equation model, RMSEA = 0.055; NFI, RFI, IFI, TLI, and CFI are all higher than or close to 0.9; CMIN/DF = 2.292, as shown in Table 3. All indexes are in line with the fitness values (Wu, 2010), indicating that the model fits well.

**Table 3:** Evaluation on goodness of model fitting (*N* = 436).

**Model**	**RMSEA**	**NFI**	**RFI**	**IFI**	**TLI**	**CFI**	**CMIN/DF**
Fitness value	< 0.08	>0.9	>0.9	>0.9	>0.9	>0.9	< 3
Default model	0.055	0.906	0.891	0.945	0.936	0.944	2.292

### Direct effect

The standardized fitting path coefficient is shown in Table 4. Through path analysis, the direct and mediating effects of the variables on entrepreneurial intention are evident. The value of the standard error of the estimated value (S.E.) in Table 4 is relatively small, indicating that the sample is closer to the total parameter. The value of critical ratio (C.R.) was used to calculate P. Clearly, the five paths indicate significance *(p* < 0.05), whereas two paths have no significance (*p* > 0.05).

**Table 4:** Coefficients of the structural model.

**Path**	**Estimate**	**Standardized estimate**	**S.E**.	**C.R**.	***P***
Personality traits < – Society support	0.210	0.258	0.069	3.052	**
Personality traits < – Family support	0.219	0.226	0.070	3.146	**
Personality traits < – Education support	−0.163	−0.145	0.063	−2.587	**
Personality traits < – Policy support	0.284	0.310	0.068	4.176	***
Entrepreneurial intention < – Personality traits	1.164	0.954	0.095	12.268	***
Entrepreneurial intention < – Family support	0.067	0.056	0.060	1.116	0.264
Entrepreneurial intention < – Education support	0.061	0.044	0.055	1.114	0.265

***P < 0.001, **P < 0.05.

#### Impact of family support on entrepreneurial intention

It was found that family support is not significantly influence entrepreneurial intention (β = 0.056, *P* = 0.264 > 0.05), which means that hypothesis 1 is not supported. This is consistent with existing results (Gimenez et al., 2015). In fact, family support and friend support are critical for college students to establish self-confidence, encouragement, and financial support (Baughn et al., 2006). This may be connected to the low willingness to start a business. There may be other reasons, which need further exploration. The characteristics of family norms, values, and resources could impact new venture creation (Aldrich and Cliff, 2003). This is especially true under the influence of Chinese Confucianism, where many parents hope their children get jobs in the government system and stable incomes and high social status. On the other hand, the risk of entrepreneurship is also higher. Moreover, regional personality plays a crucial role in the entire entrepreneurial ecosystem (Zhou et al., 2021). The college students in this study mainly come from Henan Province, which is located in central China, where the local economy relatively lags behind. Therefore, the family support turns out to be not significant.

#### The impact of education support on entrepreneurial intention

Education support does not significantly influence entrepreneurial intention (β = 0.044, *P* = 0.265 > 0.05), thus rejecting hypothesis 2. This result is unique to the Chinese experience; in fact, the training, projects, and practice have significant impacts on college students' entrepreneurial intention in existing studies. This closely relates to the recent national innovation and entrepreneurship education on college students. With the continuous growth of economics and jobs (Thurik et al., 2012), entrepreneurship education has become the focus of attention for universities, researchers, and the government (Kuratko, 2005). By contrast, college students are unsatisfied with present entrepreneurship education, which relies on short-term training or professional education before employment in China. Therefore, it is still necessary to focus on the cultivation of entrepreneurial abilities and improve the effect of entrepreneurship education in universities. In China, entrepreneurship education should go beyond traditional examination-oriented teaching styles and focus on promoting proactive personality and creativity (Hu et al., 2018).

#### The impact of personality traits on entrepreneurial intention

Personality traits influence entrepreneurial intention significantly, which supports hypothesis 3 (β = 0.954, *P* < 0.001). Self-confidence, communication ability, innovation ability, risk-taking spirit, planning, and organizational ability are decisive factors in the entrepreneurship intention of college students. The higher the risk-taking spirit and initiative, the more conducive it is to entrepreneurial intention.

### Mediating effect

For the influence of family support, education support, policy support, and social support on entrepreneurship intention, personality traits do play a fully mediating role (*p* < 0.05). Interestingly, policy support and education support do not significantly affect entrepreneurial intention directly but do have significant impacts on personality traits. Essentially, personality traits play a mediating role. Therefore, entrepreneurship intention should be based on a clear understanding of students' personality characteristics, core qualities, key competencies, and innovation.

#### The impact of family support on entrepreneurial intention

Family support significantly influences personality traits (β = 0.226, *P* < 0.05), and personality traits likewise significantly influence entrepreneurial intention. Thus, hypothesis 4 is supported. Various factors influence college students' personality traits, including optimism, self-confidence, innovativeness, risk-taking propensity, leadership (Embi et al., 2019), economic support (Suratno et al., 2021), entrepreneurial experience, self-confidence, creativity, and alertness (Baron, 2004). Along with the aforementioned analysis, personality traits indeed play a mediating role in family support on entrepreneurial intention.

#### The impact of education support on entrepreneurial intention

Education support significantly influences personality traits (β = −0.145, *P* < 0.05). The results indicate the significant effect of personality traits on entrepreneurial intention and further reveal the internal influence mechanism of education support on entrepreneurial intention. Thus, hypothesis 5 is confirmed. Entrepreneurship education promotes entrepreneurial intention by cultivating college students' self-efficacy, anticipatory thinking, creative self-efficacy, learning self-efficacy (Fuller et al., 2018), risk-taking (Zhang et al., 2015), and other important values. Thus, personality traits play a mediating role, and the relationship between entrepreneurship education and entrepreneurship intention is definitely noteworthy.

#### The influence of policy support on entrepreneurial intention

Policy support significantly affects personality traits (β = 0.310, *P* < 0.001). Although policy support does not directly influence entrepreneurial intention on a significant level; however, it does have a significant impact on entrepreneurial intention through personality traits. This verifies hypothesis 6 and reflects the mediating role of policy support. The results are also consistent with existing studies, which generally show that entrepreneurial policy and entrepreneurial intention are significantly related (Huang et al., 2021). College students generally understand national and local policies through indirect channels. Hence, the deeper the understanding of entrepreneurial policies, the stronger entrepreneurial intention is generated (Fu and He, 2020). Most especially, government support significantly influences entrepreneurial intention (Xiang and Sun, 2021). In the interview, this was found to be a relatively serious and major problem for college students—the lack of fund sources and insufficient understanding of policies led to the restrictions of entrepreneurial intention and activities. Some college students plan to be employed first and accumulated funds and experience before starting a business. The Mycos report (2020) also shows that the proportion of graduates starting their own businesses continues to rise with the extension of graduation.

#### The influence of social support on entrepreneurial intention

Social support significantly influences personality traits (β = 0.258, *P* < 0.05). In addition, personality traits influence entrepreneurial intention significantly, thus confirming hypothesis 7. Mao and Ye (2021) revealed that social capital is directly tied to the individual. Huang et al. (2021) found that the accessibility of opportunities and financial support leads to real entrepreneurial behavior—the more favorable the entrepreneurial environment perceived by college students, the likelier they are to obtain environmental resources and to improve their entrepreneurial willingness. These results also coincide with previous those of previous studies.

## Discussion

**Figure 2:**
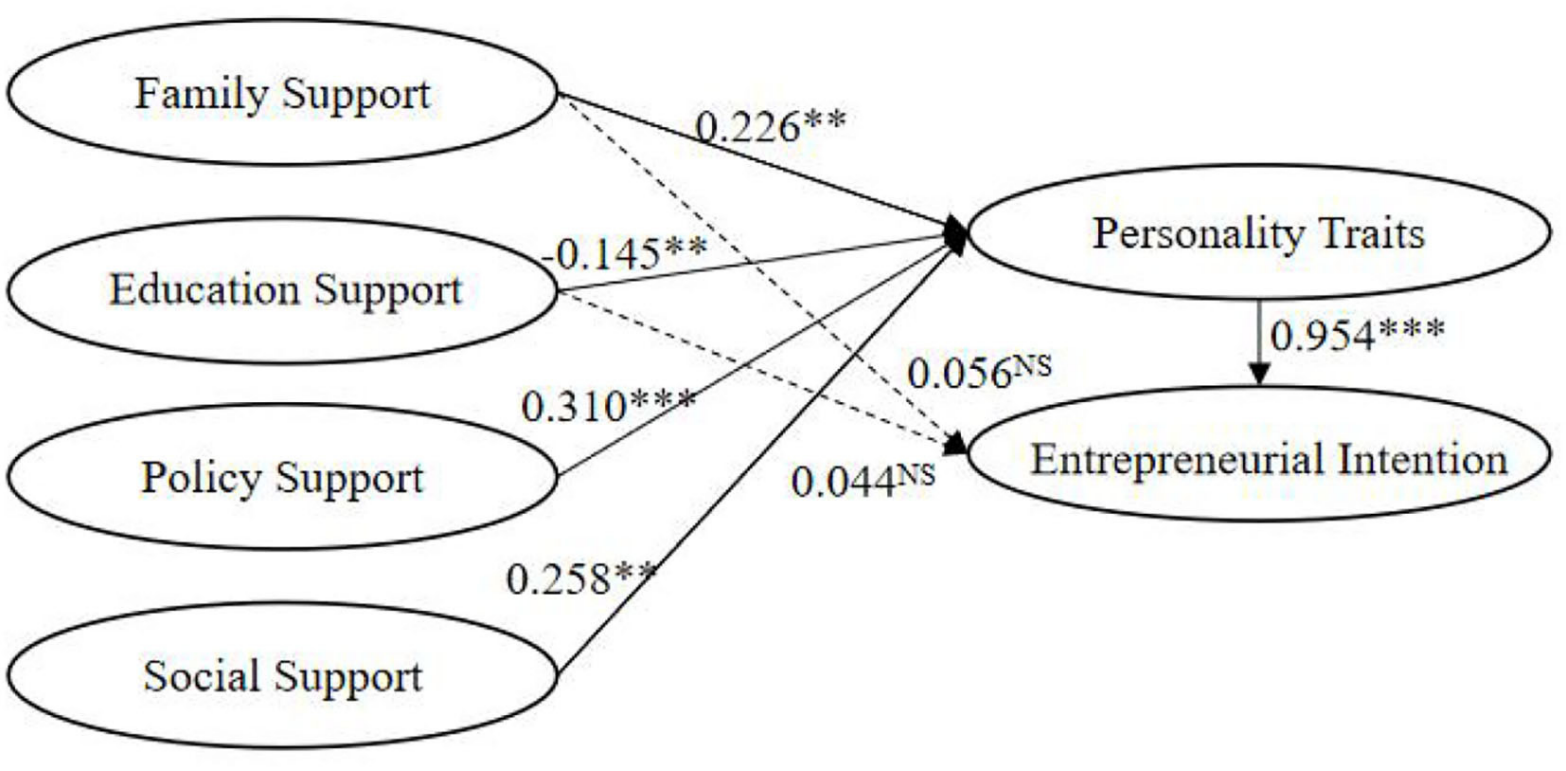
Path of SEM from research. NS, not significant; ***P < 0.001; **P < 0.05.

The path analysis of the structural equation model (see Figure 2) demonstrates that the influence of policy support, social support, educational support, and family support on entrepreneurial intention can be seen as a reflection of the mediating role of personal qualities. Given the mediating effect of personality traits, it is essential to foster college students' personality traits of entrepreneurship. Personal factors have been confirmed as stronger triggering factors than contextual ones (Sesen and Ekemen, 2020). Although some personality variables are quite stable and difficult to change, McClelland (1961) posited that some entrepreneurial qualities can be taught. Nevertheless, Moraes et al. (2018) found that some competencies such as creativity, proactivity, and propensity to risk are not fully cultivated by necessary and appropriate teaching methods.

Entrepreneurship education should reform to promote college students' entrepreneurial intention. College students' entrepreneurial intention is affected by many factors (Ajzen, 1991). Systems and mechanisms in families, society, colleges, and enterprises should be established. According to the entrepreneurial ecosystem theory (Davies et al., 2005), the following factors are in a constant feedback loop: college students' entrepreneurial intentions are generated and put into practice by creating a favorable environment for innovation and entrepreneurship and providing the cultural environment, entrepreneurial funds, entrepreneurial resources, and entrepreneurial policies. All these need the guarantee of a sound system and mechanism to lay the foundation for future entrepreneurship.

(1) Implications for college students' entrepreneurial practice: Currently, entrepreneurship is mainly promoted by the Chinese government using the top-down mode. In addition, the traditional teaching pays much attention to knowledge and professional skills, while the cultivation of students' entrepreneurship practice and ability remains lackluster. College students believe that entrepreneurship education and training are useful for entrepreneurship but have no obvious effect. College students' ability should be improved in the entrepreneurial practice. They need societal guidance; hence, universities and teachers ought to suit them to new situations and patterns. Professional education and entrepreneurship education need to be deeply integrated, and the curriculum system and teaching methods need to be reformed. This entails the creation of simulations, providing many projects, and making full use of virtual practice platforms. We should also reform the education goals, teaching methods, and learning resources coupled with other related yet necessary reforms (Shi et al., 2020). For example, case studies, group cooperative learning, visits to companies, company internships, simulations, interviews with entrepreneurs, and projects (Kuratko, 2005) are suggested in teaching. These techniques support the shift of college students from thoughts to deeds, from simulation to reality, and from private citizens to members of society. The skills, experience, and practice of business establishment and operation are also required, which include business plan writing, marketing, management, and product sales (Jamieson, 1984). Different locales can adopt unique policies in response to the various local entrepreneurial circumstances.

(2) Implications for improving college students' proactive personalities: The results of a structural equation model show that personality traits have a significant impact and play a mediating role in college students' entrepreneurial intention: Students with low-risk tendency, low innovation, and lack of adventurous personal characteristics may have low entrepreneurial intention. This outlines the need to promote college students' risk awareness, innovation ability, and initiative. First and foremost, the spirit of exploration and adventure among college students needs to be fostered. The lack of risk awareness is a major factor in the low rate of entrepreneurship. Entrepreneurial activities are adventurous (Zhang et al., 2015); therefore, the cultivation of college students' self-confidence, communication ability, innovative spirit, entrepreneurship, and creative thinking should be based on the reform of the training system and resources (Ahmad et al., 2014; Hu et al., 2018). Hence, students are encouraged to express themselves and work together as a team under a less teacher-centered model. Inspiring entrepreneurial alertness in students' cognitive processes and fostering their entrepreneurial orientation are the goals of educational activities (Solesvik et al., 2013). Second, college students' initiative personality is cultivated in entrepreneurship training, such as professional programs and the like. College students can be encouraged to participating in some projects with low investment, low risk, and easy implementation. Then, the successful experience will enhance their self-confidence and allow them to accumulate entrepreneurial experience. Through these activities, college students can improve their problem-solving abilities and creativity in relation to building their own businesses. Although COVID-19 has affected entrepreneurship, the development of the Internet has also brought new opportunities for college students to build their own enterprises. College students should make use of the opportunities presented by campus agents, self-media, online businesses, and other types of online entrepreneurship. For instance, following COVID-19, online education, retail, e-commerce, and information technology have emerged as the primary entrepreneurial sectors.

## Conclusion

This study establishes a hierarchical model of the environmental factors affecting college students' entrepreneurial intention from macro-, meso-, and micro-layers. The micro-system includes personality traits. The meso-system includes family support and education support and directly promotes entrepreneurial intention directly. The macro-system includes policy support and social support and indirectly influences entrepreneurial intention. Thus, various factors form a clear relationship. These results also provide a new perspective for further studies.

Second, the structural equation model of the environmental factors is established to clarify the impact and the relationship between internal and external factors. This supplements the existing influencing factors of entrepreneurial intention and provides a new theoretical perspective for further exploring the influencing process of internal and external environmental factors. It also explains the direct role of internal environmental factors and the indirect role of the external environment through the internal environment, which provides a certain theoretical value and the path from intention to behavior.

Third, this study empirically examined the effects of policy support, social support, family support, education support, and personality traits on entrepreneurial intention through questionnaires and data analysis. Ultimately, it tried to explore two research questions: (1) What are the factors that influence college students' entrepreneurial intention? (2) Do personality traits play the mediating effect of environmental factors on entrepreneurial intention? Based on the survey and results, personality traits have significant impacts on college students' entrepreneurial intention, while family support, education support, policy support, and social support do not. Moreover, all the environmental factors (society support, family support, education support, and policy support) have significant impacts on personality traits. Hence, hypotheses H3, H4, H5, H6, and H7 are supported, while H1 and H2 are not confirmed. Personality traits play a mediating role in the environmental factors on entrepreneurial intention.

One limitation of this study is that the questionnaires were mainly implemented in Henan Province, thus restricting generalizability. Future studies could enlarge the survey scope in response. The reasons for some results need to be revealed in combination with interviews. There remains a large gap between entrepreneurial intention and behavior, which is also worthy of depth study. The investigation of the goal and ability is also made easier by failure entrepreneurship, a new area of study in entrepreneurship. In order to analyze the variations in experience between failure and success, we should further investigate the causes of entrepreneurial failure.

## Data availability statement

The raw data supporting the conclusions of this article will be made available by the authors, without undue reservation.

## Ethics statement

Ethical review and approval were not required for the study on human participants in accordance with the local legislation and institutional requirements. Written informed consent from the patients/participants or patients/participants legal guardian/next of kin was not required to participate in this study in accordance with the national legislation and the institutional requirements.

## Author contributions

JJ contributed to the design of the study, data extraction, data analysis, interpretation of the results, and manuscript. JX reviewed and edited the manuscript. XY contributed to the design of the study, literature, and writing of the manuscript. JH was involved in the data collection process. All authors have read and agreed to the submitted version of the manuscript.
